# Real-time acid production and extracellular matrix formation in mature biofilms of three *Streptococcus mutans* strains with special reference to xylitol

**DOI:** 10.1016/j.bioflm.2024.100219

**Published:** 2024-08-28

**Authors:** Henna Ikäläinen, Camilo Guzman, Markku Saari, Eva Söderling, Vuokko Loimaranta

**Affiliations:** a Institute of Dentistry, University of Turku, Lemminkaisenkatu 2, 20520, Turku, Finland; b Cell Imaging and Cytometry Core, Turku Bioscience, University of Turku and Åbo Akademi University, Turku, Finland; c Euro-Bioimaging ERIC, Turku, Finland

**Keywords:** Biofilm, *Streptococcus mutans*, Real-time assay, pH, Xylitol

## Abstract

**Background:**

Acidogenicity and production of an extracellular matrix (ECM) are important virulence factors for the dental caries-associated bacteria, such as *Streptococcus mutans,* that live in biofilms on tooth surface. The ECM protects the bacteria from the flushing and buffering effects of saliva resulting in highly acidic microenvironments inside the biofilm.

**Materials and methods:**

In this *in vitro* study, we applied real-time assays to follow biofilm formation and pH decrease in a growth medium and saliva by three *S. mutans* strains, as well as acid neutralization inside the mature biofilm. Results were compared with the biofilm composition. Effects of a non-fermentable polyol, xylitol, on acid production and acid neutralization in mature biofilms were evaluated by real-time pH measurements and confocal microscopy.

**Results:**

Combination of real-time pH measurements with biofilm accumulation assays revealed growth media dependent differences in the pH decrease and biofilm accumulation, as well as strain differences in acid production and biofilm formation but not in the buffer diffusion through ECM. The presence of xylitol reduced the pH drop during biofilm formation of all strains. In addition, with strain Ingbritt xylitol reduced the amount of ECM in biofilm, which increased the rate of acid neutralization inside the biofilm after buffer exposure.

**Conclusion:**

Our results stress the importance of biofilm matrix in creating the acidic environment inside a *S. mutans* biofilm, especially in the presence of saliva. In addition, our results suggest a novel mechanism of xylitol action. The observed increase in the permeability of the *S. mutans* ECM after xylitol exposure may allow acid-neutralizing saliva to reach deeper layer of the biofilms and thus, in part, explain previous clinical observations of reduced plaque acidogenicity after frequent xylitol use.

## 1 Introduction

Dental caries is a highly prevalent infectious, chronic disease that affects most of the human population at some stage [1]. Caries is initiated by bacterial-produced acids that demineralize the tooth enamel. Bacteria accumulate on the tooth surface and form biofilm, also known as dental plaque. In the biofilm microbial cells are surrounded by extracellular matrix (ECM) polymers such as secreted exopolysaccharides (EPS), proteins and nucleic acids (eDNA) [2]; [3,4]. The ECM composition is important for dental plaque accumulation but also for the ability of environmental components to penetrate the biofilm. In the cariogenic biofilm, the ECM prevents diffusion of bacterial produced acids out from the biofilm while the salivary buffering components do not reach the inner parts of the biofilm structure. Thus, the microenvironment inside the biofilm can remain highly acidic despite the continuous flow of saliva and its high buffering capacity.

In the oral biofilm, mutans streptococci are efficient acid producers, and also major producers of biofilm matrix polymers, making them prominent cariogenic bacteria in the oral cavity [5,6]). However, strain dependent differences in virulence are well acknowledged e.g. by Esberg et al. [7]; [8], stressing the importance of the behavior comparison between different strains.

Xylitol, a naturally occurring five-carbon polyol sweetener, is not fermented by oral micro-organisms and it appears to have specific, beneficial effects on oral health and also other health benefits [9]; [10]. Habitual consumption of xylitol, especially in the form of chewing gum, has reduced caries occurrence in studies with a high or moderate caries level at study baseline [11]. Systematic reviews have suggested that xylitol consumption reduces both the levels of mutans streptococci and the amount of dental plaque [12]; [[13], [14], [15]]. Several studies have also reported that xylitol consumption reduces plaque acidogenicity, e.g. Ref. [16]. Despite the abundant research published, the mechanisms of action of xylitol are not fully understood. A less adhesive plaque due to a decrease in the counts of mutans streptococci and/or reduced amounts of EPS in the plaque have been suggested to explain the reduction in the amount of plaque [9]. The growth of planktonic cells of mutans streptococci is inhibited by even low concentrations of xylitol [17]. However, *in vitro* biofilm studies have resulted in contradictory results both on the effects of xylitol on counts of mutans streptococci and EPS production [18]; [19].

Despite the acknowledged importance of the acidity inside the biofilm, there are not many methods to measure pH inside the biofilm without disturbing its natural structure. Very small pH electrodes have allowed to measure pH inside the dental plaque and artificial biofilms, but they disrupt the matrix and are prone to disturbances [[20], [21], [22]]. The pH changes in intact *in vitro* biofilms have been measured by fluorescent reporters to follow the expression of pH-sensitive genes [23]; [24] or by pH-sensitive fluorophores and confocal microscopy ([25]; 2020; [26]). These applications have widened our understanding of pH dynamics and regulation inside the biofilm, but their use is limited by high labor intensity and the need for genetically modified bacteria or advanced imaging techniques and analysis programs.

The bioprocessing industry is continuously increasing and online measurements of the processes are crucial. Therefore novel applications are developed and, for example, the field of optical sensors has been under extensive studies. Indeed, the immobilized pH-sensitive sensors are successfully used to monitor real-time pH changes in bioprocesses and in eukaryotic cell cultures (e.g. Ref. [27]; [28]). Such sensors are easy to use and they can be connected to computers for automatic recording of pH changes. In this study, we tested the suitability of such integrated optical pH sensors to measure real-time pH changes during biofilm formation by three *S. mutans* strains in rich growth media or saliva, and to measure the dynamics of acid neutralization inside the mature biofilms after buffer exposure. The results were correlated to the ECM composition. With the *S. mutans* strain Ingbritt, the above variables were studied for the effects of xylitol added to the growth media and compared to results obtained by pH sensitive fluorescent probes in confocal microscopy.

## 2 Materials and methods

### 2.1 Bacterial strains and growth conditions

Three *S. mutans* strains, *S. mutans* NCTC 10449 and *S. mutans* Ingbritt (IB), and one clinical isolate *S. mutans* CI2366 [17] were used. To obtain an inocula for the assays, the bacteria were grown overnight in Brain Heart Infusion Broth (BHI, Becton Dickinson, France) at 37 °C. On the day of the experiment, the bacteria were washed once with 0.9 % NaCl and suspended in NaCl to obtain OD_550_ = 0.05.

### 2.2 Biofilm formation

The biofilm reaction mixtures contained 1 vol of either BHI or human saliva as a growth medium, 1/2 vol of sucrose (final conc. 1 %, dissolved in 0.9 % NaCl) and 1/2 vol bacterial suspension. Stimulated whole saliva was collected from six individuals, pooled and pasteurized as described [29].

#### 2.2.1 Biofilm on the electronic microtiter plates

Real-time biofilm accumulation was measured with an xCelligence RTCA DP instrument (ACEA Biosciences Inc, CA, USA, [30,31]). The attachment of bacteria on an electronic microtiter plate (E-plate) alters the measured impedance, expressed as an arbitrary unit called cell index (CI), and the changes in impedance can be followed in real time. The instrument was placed in a CO_2_ incubator with a temperature of 37 °C, and the biofilm formation was measured in 15 min intervals for 24 h. The total volume in the well was 200 μl and experiments were made at least in duplicates and repeated at least once.

#### 2.2.2 Biofilm on the pH sensor plates

Biofilms were allowed to form on 24 well SDR HydroDish plates (PreSens Inc, Regensburg, Germany) in a total volume of 2 ml per well. Sensor dishes contain pre-calibrated pH sensors integrated at the bottom of each well and are read through the transparent bottom of the dish by PreSens Sensor Dish Reader (PreSens Inc). The fiberoptic pH sensors have a measurement range from 5.0 to 8.5. The plates and the reader were placed in a CO_2_ incubator at 37 °C, and the pH changes were measured in 15 min intervals for 24 h. The experiments were made at least in duplicates and repeated at least once.

### 2.3 Buffer diffusion

To test the buffer diffusion through mature biofilms, the biofilms were allowed to form on HydroDish plates as described in 2.2.2 and after 24 h the medium in the wells was changed to fresh medium (BHI+1 % sucrose). After additional 24 h the plate with the biofilm was transferred to a cold room (8 °C) for 1 h to reduce bacterial metabolism during the diffusion assay, which was performed in the cold room. The medium was removed and the wells were briefly rinsed with 2 ml of PBS, pH 7.4. A fresh 2 ml of PBS was added to the biofilm and the pH changes were followed at 1 min intervals. At 5 min, 15 min and 45 min aliquots of 300 μl were taken from the surface of the liquid, and their pH was measured with a pH electrode. The experiments were made at least in duplicates and repeated once.

### 2.4 Biofilm composition analysis

For the composition analysis, 48-h biofilms were grown on 24 well plates (Costar™) as described in section 2.3. Before collecting biofilms for analysis they were carefully washed twice with 0.9 % NaCl. For the biofilm collection, 200 μl of NaCl was added in each well and the biofilm was detached using a micro brush (Quick-Stick, Dentsolv AB, Sweden) as previously described [18].

#### 2.4.1 eDNA

Biofilm samples were vortexed vigorously to disrupt the biofilm structure and bacterial cells were removed by centrifugation 5 000*g* for 5 min at ambient temperature. The eDNA was measured from the supernatant (diluted 1:10) with Quant-iT™ PicoGreen™ Assay Kit (Invitrogen, MA, USA) according to the manufacturer's protocol.

#### 2.4.2 Extracellular polysaccharide

The biofilms were analyzed as such to measure the total polysaccharides or mixed first with 100 μl of NaCl and incubated for 1 h at 37 °C. After incubation, the samples were centrifuged for 10 000 *g,* 10 min and the non-soluble polysaccharides measured from the pellet [32]. The total polysaccharide amount in the biofilms was measured by the Anthrone method [32,33] with some modifications. Briefly, the collected biofilm was mixed with an equal volume of 0.8 M NaOH and centrifuged (10 000*g*, 10 min). The supernatant was diluted 1:5 or 1:7 with distilled water and used for analysis. An aliquot of 100 μl of the sample was mixed with 300 μl anthrone-sulfuric acid reagent (10 mg of anthrone per 10 ml of sulfuric acid) on ice. After 10-min incubation on ice samples were boiled for 20 min. Absorbance at 620 nm was measured after samples were cooled to room temperature (20–22 °C). The obtained absorbance was compared to the standard curve derived from samples with a known amount of dextrose. Polysaccharides were measured in duplicates from 4 replicates, and the amount of carbohydrates in the biofilm suspension (μg/ml) was calculated.

#### 2.4.3 Colony forming units (CFU)

Before analysis the collected biofilm samples were placed on ice and sonicated 2 × 5 s. Samples were vigorously vortexed before, after as well as between the sonications. The CFU-values were obtained by serially diluting the collected biofilm sample and plating on BHI-agar plates.

### 2.5 Effects of xylitol

Effects of xylitol on *S. mutans* acid production was explored with all three strains. The bacteria were grown overnight in BHI medium, washed and suspended in fresh BHI medium (OD_550_ = 0.05). Media supplemented with 1 % sucrose or 1 % sucrose and 4 % xylitol (w/v, final conc) were prepared by dissolving the supplements in the BHI medium (instead of NaCl as done in step 2.2) and filter sterilized. Biofilms were allowed to accumulate in 24-well HydroDish plates in BHI supplemented with 1 % sucrose or 1 % sucrose and 4 % xylitol and the acid production was measured as described above. The strain IB was selected further studies for pH neutralization inside the biofilms as well as the eDNA, EPS and CFU measurements which were measured as described above.

For comparison, the pH neutralization of *S. mutans* IB biofilm was additionally measured with pH-sensitive fluorescent probes and confocal microscopy as described earlier [24]. Biofilms were allowed to accumulate on glass bottom petri dishes (35 mm Petri dish, MatTek co, MA, USA) in BHI supplemented with 1 % sucrose or 1 % sucrose and 4 % xylitol (w/v, final conc) as described above, except that 2 μl Lysosensor yellow/blue dextran (10 000 Mw, Molecular probes)/3 ml medium was added, both in the beginning and when fresh medium was added. Lysosensor has a dual-emission spectral peak (452 nm and 521 nm), and the ratio between the fluorescence intensity of these two peaks is pH-dependent.

Microscopy measurements were done at room temperature with a Carl Zeiss LSM880 laser scanning confocal microscope running ZEN 2.3 SP1 Black Edition software (Carl Zeiss GmbH, Jena, Germany). A 20x/0.8 Zeiss Plan-Apochromat objective was used for imaging. Excitation of the pH sensor was done with a 405 nm laser, and emission ranges of 410–482 nm and 499–597 nm were collected simultaneously on a spectral GaAsP detector with a gain of 750 V. The pinhole was set to 1.11 AU corresponding 1.5 μm thick optical section, and 3D image stacks were imaged through the total volume of the biofilms with a step size of 1.0 μm. Image frames of 1024x1024 (pixel size 0.415 μm) were acquired by bidirectional scanning with the line averaging of 2, pixel dwell time 0.51 μs (frame scan time 1.26 s), from 3 to 5 randomly chosen locations of the sample (XY positions), every 90 s for 20–30 min by using a Carl Zeiss Definite Focus drift correction hardware. The fluorescence intensity of both emission wavelengths and the ratio of fluorescent intensity within each biofilm image were measured using Image J 1.44 and its calculation tools (Rasband, W.S., ImageJ, U. S. National Institutes of Health, Bethesda, Maryland, USA, https://imagej.net/ij/, 1997–2018).

### 2.6 Statistics

The differences between groups were compared with *Student's t-*test. The *p*-value <0.05 was considered as significant.

## 3 Results

### 3.1 Biofilm on the electronic microtiter plates

**Fig. 1:**
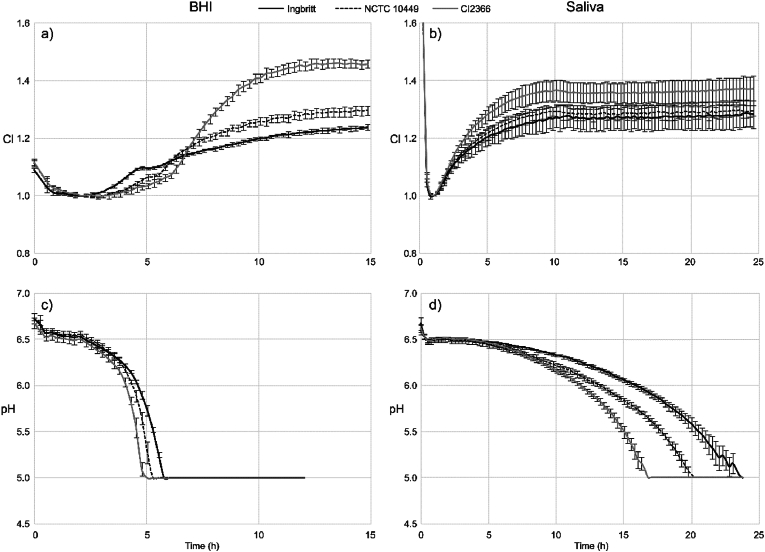
Biofilm accumulation and pH changes in Brain Heart Infusion (BHI) media and in saliva of *S. mutans* strains Ingbritt, NCTC 10449 and CI2366. Overnight grown bacteria were washed and supended in NaCl solution and added into indicated growth media. Biofilm accumulation was followed by impedance based measurement (a,b) and pH changes measured by integrated fiberoptic pH sensors (c,d). CI: cell index. Mean ± SD.

The rate of biofilm accumulation differed in sucrose-supplemented BHI and saliva, being faster in saliva (Fig. 1a and b). The highest biofilm formation was recorded for *S. mutans* CI2366 in both media.

### 3.2 pH changes

The strain CI2366 induced the fastest drop in pH in both media. In sucrose-supplemented BHI the fastest pH decrease was recorded between 4 and 6 h (Fig. 1c), when the biofilm accumulation was only in early phases (Fig. 1a). In sucrose-supplemented saliva, the pH drop was noted at much later time points, and the biofilm accumulation was stabilized before the pH decrease became evident (Fig. 1b and d). The BHI medium contains 0.2 % glucose and all strains produced acids from the glucose available in the BHI medium but no acid production was measured in saliva without the sucrose supplementation.

**Fig. 2:**
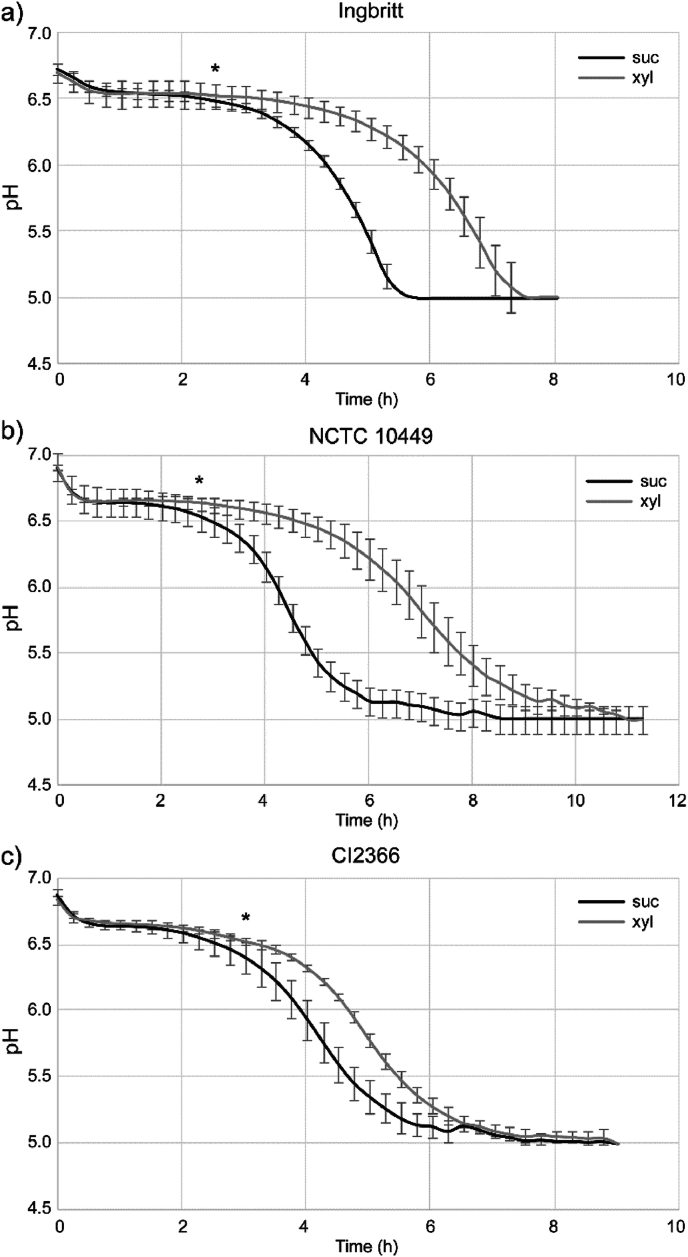
*S. mutans* strains were grown in BHI -sucrose medium with and without xylitol and pH changes monitored by real-time with fiberoptic pH sensors intergated in the growth plate. * indicates the first time point for significant difference Mean ± SD.

Addition of xylitol to the sucrose-supplemented BHI medium reduced the pH drop during the biofilm formation of all strains (Fig. 2.)

### 3.3 Buffer exposure

**Fig. 3:**
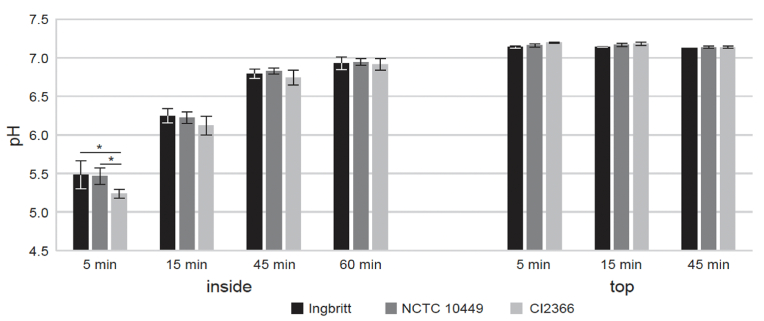
Changes of the pH after exposing the biofilm to PBS buffer. The mature biofilms of three *S. mutans* strains Ingbritt, NCTC10449 and CI2366 were washed and exposed to neutral buffer. The pH changes inside the biofilm were measured by integrated pH sensors. At indicated times aliquots of the buffer in the wells were withdrawn and pH measured with a pH electrode. Inside: the pH inside the biofilm, top: the pH of the buffer on the top of the biofilm.

To measure the permeability of the biofilm ECM, biofilms were allowed to grow on the wells of Sensor plates and exposed to PBS. The buffer diffusion through the biofilm matrix was followed by measuring pH changes inside the biofilm. The pH neutralization was very similar in biofilms formed by all the tested strains, except that at the early time points (up to 5min) the pH inside the biofilm formed by *S. mutans* CI2366 was significantly lower than the pH inside the biofilms formed by the two other strains (Fig. 3). It took about an hour to reach a neutral pH inside the biofilms. The pH of the buffer on the top of the biofilm remained relatively constant (7.1–7.3) throughout the assay (Fig. 3).

When bacteria were grown in BHI medium without added sucrose, thin biofilms/layers of precipitated bacteria were accumulated on the surface of the wells. In those wells the pH was neutralized within 20 min and no differences were observed between the strains.

**Fig. 4:**
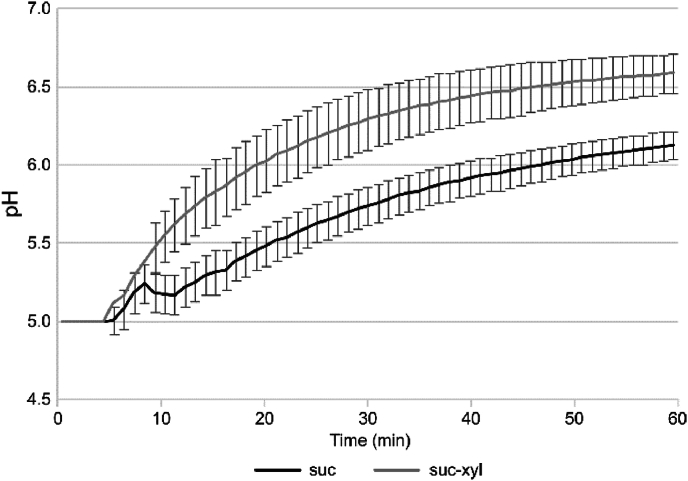
The pH neutralization inside the mature biofilm formed by *S. mutans* Ingbritt in the presence (suc-xyl) or absence (suc) of xylitol after buffer exposure. The biofilms were allowed to accumulate in the presence and absence of the xylitol. After 48 h the biofilms were washed and exposed to neutral buffer and the pH was followed by integrated pH sensors. Mean ± SD.

The biofilms were allowed to accumulate w/wo added xylitol in the sucrose-supplemented medium for 48-h before exposure to the PBS buffer. The pH inside the xylitol biofilm was neutralized at a significantly faster rate compared to the control biofilm (Fig. 4).

### 3.4 Confocal microscopy

**Fig. 5:**
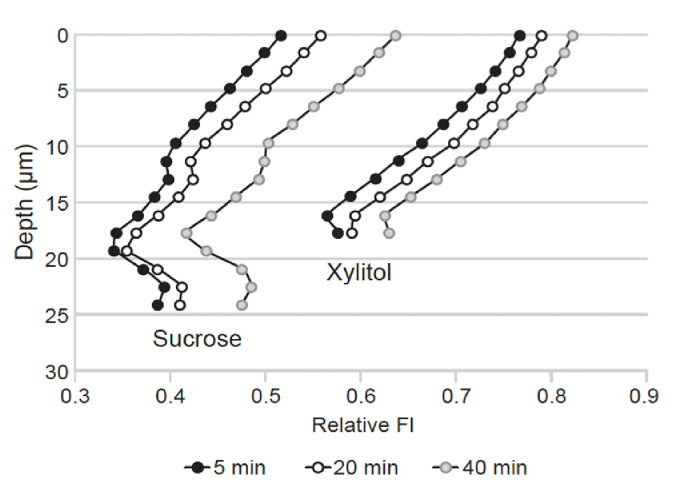
The pH neutralization inside the mature biofilm formed in growth medium with and without xylitol supplemented with pH sensitive fluorophore. The biofilms were exposed to neutral buffer and the pH changes were measured by confocal microscopy as changes in fluorescence ratio of the fluorophore in different levels of the biofilm. Relative Fl: fluorescence 452/521, higher ratio reflects higher pH.

The biofilm of *S. mutans* Ingbritt grown in the sucrose-supplemented medium was thicker than the biofilm grown with added xylitol (24 μm vs 18 μm; Fig. 5). In both the control and xylitol biofilms, the pH (measured as change in fluorescence ratio, 450/530 nm) was lower the deeper inside the biofilm it was measured. After buffer exposure, the pH increased in all levels of the biofilm and, similarly to HydroDish assay, the pH remained lower in the biofilm without xylitol (Fig. 5).

### 3.5 Biofilm composition analysis

The number of viable bacteria and the composition of the 48-h biofilms grown in the sucrose-supplemented medium were evaluated by plate counting. The number of live bacteria was similar in the biofilms of the three *S. mutans* strains (Table 1). The amount of extracellular carbohydrates differed slightly between strains. The biofilm formed by strain CI2366 contained highest amount of non-soluble extracellular carbohydrates while the amount of the water-soluble carbohydrates was significantly highest in biofilms formed by strain NCTC 10449. Also the amount of eDNA in ECM differed between strains, being highest in the *S. mutans* IB biofilm (Table 1).

**Table 1:** Composition of biofilms formed by different S. mutans strains in BHI 1 % sucrose medium.

	Extracellular carbohydrate (ug/ml)			
Strain	Non-soluble	Water soluble	eDNA (ug/ml)	logCFU/ml
Ingbritt^#^	97 ± 6	35 ± 29	11.2 ± 0.6	7.6 ± 0.3
NCTC 10449^#^	105 ± 10	89 ± 22^b^^,^^c^	7.1 ± 0.6^b^^,^^c^	8.2 ± 0.2
CI2366^#^	113 ± 11^a^	44 ± 18	10.6 ± 0.4^a^	7.9 ± 0.1

a significant difference to strain Ingbritt p < 0.05.

b significant difference to strain CI2366, p < 0.01.

c significant difference to strain Ingbritt, p < 0.001.

**Fig. 6:**
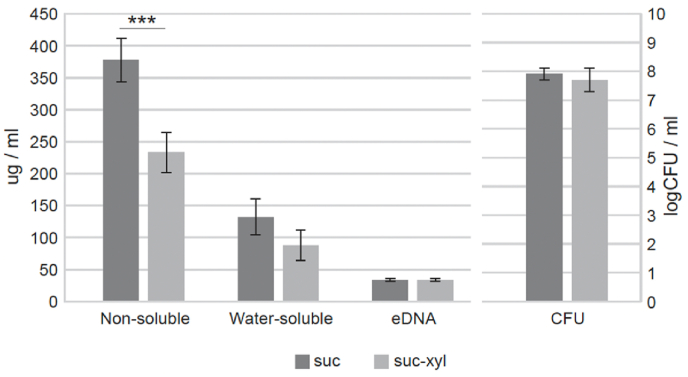
The effect of xylitol on biofilm composition. *S. mutans* Ingbritt biofilms were allowed to accumulate in BHI-sucrose growth medium with and without xylitol. The amount of Non-soluble and water -soluble exctracellular carbohydrates as well as extracellular DNA (eDNA) and the amount of bacteria (colony forming units, CFU) were measured.

The presence of xylitol in the sucrose-supplemented growth medium reduced significantly the amount of non-soluble extracellular carbohydrates in the biofilm matrix. Also the water soluble carbohydrates were reduced by 33 %, but this change was not statistically significant. No effect was found on the amount of eDNA or the amount of live bacteria (Fig. 6).

## 4 Discussion

We studied the biofilm formation by three *S. mutans* strains in two different media, in a rich artificial growth medium BHI and human saliva, a more natural environment of *S. mutans.* All strains formed biofilms in the presence of sucrose and produced acids from glucose and sucrose, but the biofilm accumulation and the acid production differed between strains and between the media, as shown also earlier e.g. Ref. [34]; [31]. The two real-time assays revealed interesting links between the pH changes and biofilm accumulation. When bacteria were grown in BHI supplemented with sucrose, the pH 5, the lowest measurable pH of the fibreoptic sensor, was reached within 5 h while the biofilm mass accumulation was strongest at much later time points. Thus, it is likely that the measured pH values reflected the pH in the growth medium because no biofilm was yet formed. In the saliva, however, the pH decrease was noted at much later time points, and only after the biofilm accumulation was evident. It remains unclear whether the noted difference between BHI and saliva is due to higher acid production in BHI or better buffering capacity of saliva. *In vivo,* the regulation of the intra-oral pH relies on the neutralizing effects of saliva and saliva has a strong buffering capacity attributed to its bicarbonate content, with smaller contributions from salivary phosphates, proteins and other components that efficiently neutralize acids in the oral cavity [35]. It thus is likely that the pH decrease in saliva was possible only after enough biofilm matrix was accumulated to separate the buffering properties of the saliva from the bacterial-secreted acids. This suggests that the acids produced by *S. mutans* from sucrose can be efficiently buffered by saliva, but when biofilm matrix, or dental plaque, is accumulated the buffering molecules of saliva do not penetrate the matrix and acids may accumulate inside the biofilm.

Hwang et al [24] showed, using pH-responsive fluorophores and confocal microscopy, that incubation of *S. mutans* biofilm in a neutral pH buffer neutralizes the upper layers of the biofilm colony, but the bottom layer of the biofilm remains acidic for a prolonged time. In line with that, we could see very slow kinetics in acid neutralization in the bottom of mature biofilms after buffer exposure. Though the detailed mechanisms are unclear, the EPS are suggested to be important in the creation of an acidic environment inside the biofilm by controlling the diffusion of charged molecules into and out of the biofilm [24,36,37], but also contradictory results have been published [38]; [39]. In our study, we saw no difference in pH neutralization inside the mature biofilms produced by the three *S. mutans* strains despite significant differences in the extracellular carbohydrate amounts in the biofilms. Molecular traffic through the matrix is likely to be a sum of several components, such as secreted exopolysaccharides, proteins and nucleic acids (eDNA) [2]; [3,4], as well as the 3D structure of the matrix [25]. The clinical isolate CI2366 showed the highest biofilm accumulation in the impedance assay and also in the composition analysis the strain produced the highest amounts of non -soluble extracellular carbohydrates into the matrix of the 48-h biofilm. The impedance change is dependent on the amount of attached material, but also on the composition of the attached material and the tightness of the attachment so that sedimented bacteria give rise to negligible signal (e.g. Ref. [30]). Thus, it is tempting to speculate that the biofilm formed by strain CI2366 is more compact and/or more tightly attached on the surface. In addition to proteins and polysaccharides, eDNA is a major component of the extracellular matrix of bacterial biofilms [3]. eDNA in the *S. mutans* biofilm is suggested to relate to the number of live bacteria in the biofilm [40], but in our study the eDNA amount appeared to be strain specific.

The acid accumulation inside the biofilm, despite the near neutral pH environment found in the oral cavity, serves as a major virulence factor for the development of dental caries. Fiberoptic pH sensors can be utilized in measuring real-time pH changes during biofilm accumulation and inside a mature biofilm. Obtained results of the pH changes on sensor plates were comparable to results measured by confocal microscopy and pH-sensitive fluorescent labels. The fiberoptic sensor method was less laborious to perform, could be done in 24-well plate format and did not require sophisticated calculation methods and imaging instruments to record pH differences making it a suitable method for, for example, screening purposes. Obtained results revealed differences in acid production of different *S. mutans* strains, and we also showed that the presence of xylitol reduces the pH decrease and modifies the biofilm of *S. mutans* Ingbritt in a way that affects the acid neutralization inside the mature biofilm matrix. It has been earlier demonstrated that xylitol decreased acid production and growth of mutans streptococci in the presence of sucrose and glucose in strictly anaerobic conditions like those in deep layers of dental biofilm [41]. In that study only the final pH of the culture could be measured. The possibility to measure real-time pH-changes allows to follow pH-changes induced by various therapeutic substances of products for oral care affecting the integrity of the biofilm. A disadvantage of the present method is that the lowest pH that can be measured is pH 5. However, the pH 5.5 is considered as a critical pH for enamel dissolution, and thus the obtained pH values can be considered sufficient to evaluate the risk for oral health.

Cariogenic biofilms are comprised of mixed microbiota bound by a common matrix. In this study we focused on the ECM and pH of a single species biofilm of the caries-associated oral pathogen *S. mutans* that is an important ECM producer in oral biofilms, but it remains to be determined how the acidicity and ECM characteristics are modified by environmental factors, such as saliva and available carbohydrates, in a more complex biofilm where several bacteria and their byproducts influence the environment. For such studies the possibility to easily follow pH changes inside the biofilm in real-time is an interesting addition to the assay catalogue.

In clinical trials habitual xylitol consumption has decreased counts of mutans streptococci [12]; [13]. Biofilm studies, however, show contradictory findings on the effects of xylitol on the levels of *S. mutans* [18]; [19,31]. The results of these studies are largely influenced by methodological differences in the approaches. Apparently in early stages of biofilm formation xylitol has reduced counts of *S. mutans* but in mature biofilms this effect was not observed [18]; [31]. Interestingly, in one study xylitol inhibited in a dose-dependent fashion *S. mutans* biofilm formation and altered the biofilm architecture but only in sucrose-free conditions [42]. In the present study xylitol added to the sucrose-supplemented medium did not decrease the numbers of *S. mutans* when measured from the 48-h biofilm. This result is thus in agreement with earlier studies showing that mature “xylitol biofilms” of S*. mutans* grown in the presence of sucrose do not show reduced counts of bacteria [18]; [31,42].

Even though the numbers of *S. mutans* and the eDNA content in the mature biofilms were not influenced by the presence of xylitol the amount of non-soluble extracellular carbohydrates in the biofilm matrix decreased significantly. A few clinical trials have suggested that xylitol could decrease the content of insoluble glucans in the dental plaque [43]; [44]. Long-term, habitual xylitol consumers were also reported to have low plaque levels on their tooth surfaces indicating reduced plaque adhesion to the teeth [45]. Our results support the idea that rather a decrease in the extracellular carbohydrates than in the counts of mutans streptococci would explain the reductions in the amount of plaque reported in clinical trials [14,15]. The decreases in the extracellular carbohydrates found in this study resulted thinner biofilm and had a profound effect on the permeability of the extracellular matrix of the biofilm, as demonstrated in the buffer diffusion experiments. It is likely that the changes not only in the amount of the ECM but also its composition enabled faster diffusion of the acids produced. This finding could at least partly explain the reduction in plaque acidogenicity of xylitol consumers in clinical trials [16]. Our above findings may thus shed some new light on the mechanisms of action of xylitol. Interestingly, there are studies indicating that xylitol has shown structural disruption of ECM in biofilms of bacteria the growth of which is not inhibited by xylitol. For example, the ability of xylitol to disrupt the ECM of *Pseudomonas aeruginosa* has been used in the treatment of chronic wound infections: xylitol has enabled silver and lactoferrin to reach the deep layers of wound biofilms [46]; [47]. This suggests that the xylitol-induced changes in the permeability of the ECM of the dental plaque in xylitol consumers could enhance the effect of therapeutic substances included in various oral hygiene products. Further studies are need to test such hypothesis.

## Ethics approval and consent to participate

Not applicable.

## Acknowledgements

This research did not receive any specific grant from funding agencies in the public, commercial, or not-for-profit sectors. The support of Biocenter Finland and Euro-BioImaging (https://www.eurobioimaging.eu) for providing access to imaging technologies and services via the Finnish Advanced Microscopy Node (Turku, Finland) as well as the skillful technical assistance of Ms Oona Hällfors is gratefully acknowledged.

## Availability of data and materials

Data will be made available on request.

## CRediT authorship contribution statement

**Henna Ikäläinen:** Writing – original draft, Visualization, Investigation, Formal analysis. **Camilo Guzman:** Writing – review & editing, Validation, Investigation, Formal analysis. **Markku Saari:** Writing – review & editing, Methodology, Investigation, Formal analysis. **Eva Söderling:** Writing – review & editing, Writing – original draft, Visualization, Validation, Data curation. **Vuokko Loimaranta:** Writing – review & editing, Validation, Supervision, Methodology, Data curation, Conceptualization.

## Declaration of competing interest

The authors declare that they have no known competing financial interests or personal relationships that could have appeared to influence the work reported in this paper.
